# Role of ultrasound and inflammatory factors in the management of pediatric hip joint effusion

**DOI:** 10.1186/s12969-023-00922-8

**Published:** 2023-12-19

**Authors:** Seyed Ali Alamdaran, Mohadeseh Taheri-nezhad, Ahmad Nouri, Farzaneh Khoroushi, Mohammad Hasan Aalami, Abdoreza Malek, Arezou Mohtasham, Mohamadreza Alizadeh

**Affiliations:** 1 Mashhad University of Medical Sciences, Mashhad, Iran; 2 Faculty of Medicine, Semnan University of medical Sciences, Semnan, Iran; 3 Tehran University of medical science, Tehran, Iran

**Keywords:** Hip, Joint effusion, Arthritis Ultrasound

## Abstract

**Background:**

Septic arthritis is an important differential diagnosis of hip joint pain. Joint aspiration analysis is a necessary diagnostic measure for septic arthritis. In order to reduce the need for joint aspiration, we compared the combination of ultrasound findings and laboratory findings to separate septic arthritis from reactive arthritis.

**Methods:**

Children aged < 14 years who were referred to Akbar pediatric hospital in 2020–2022 with hip pain or limping were included in this longitudinal study. Participants underwent ultrasound examinations of the hip and blood samples were obtained from them. After confirming an effusion, dependent on patient status and clinical diagnosis, one of the following approaches was recommended; the close follow-up, or the ultrasound-guided aspiration of the hip joint effusion, and or arthrotomy. The various ultrasound and laboratory were documented. Data were analyzed and P < 0.001 being considered statistically significant.

**Results:**

Overall, 115 patients with a mean age of 3.43 ± 5.76 years, 46 of whom were girls, were studied. The final diagnosis in 23 cases (20.0%) was septic arthritis and 92 (80.0%) had reactive arthritis. C-reactive protein (CRP) and The erythrocyte sedimentation rate (ESR) unlike aspirate volume, effusion volume measured on ultrasound, capsule thickness, total thickness, and recorded capsule-to-effusion ratio were significantly higher in patients with septic arthritis (P < 0.001). There was a significant agreement between the volume of measured fluid in the anterior recess and the volume of aspirated fluid (2.5 times, P < 0.001). Septic arthritis was not observed in any of the patients with effusion volume in anterior recess less than 0.5 cc and ESR less than 40 mm/hr or CRP less than 15 mg/L.

**Conclusion:**

Since septic arthritis was not observed in any of the patients with effusion volume < 0.5 cc and normal inflammatory factors (ESR or CRP), conservative management and close follow-up can be recommended in these patients instead of joint fluid aspiration.

**Supplementary Information:**

The online version contains supplementary material available at 10.1186/s12969-023-00922-8.

## Introduction

Hip joint effusion is one of the most commonly encountered complaints by general practitioners and orthopedists. The underlying pathologies of such disorders vary greatly in importance, as they may range from the rather insignificant transient synovitis and reactive arthritis to more serious conditions such as septic arthritis. Transient synovitis is the main cause of non-traumatic hip pathologies [1]. However, symptoms such as high fever are indicative of potentially more life-threatening conditions that require early interventions. Under poor treatment situations, septic arthritis leads to permeant joint damage or even death [2–4].

Contrary to septic arthritis, which is an inflammatory joint condition secondary to bacterial inoculation of the joint via direct trauma or hematogenous spread, reactive arthritis and transient synovitis are inflammatory disorders caused by a remote infection with risk factors such as upper respiratory, gastrointestinal, or urinary infections [5]. Reactive arthritis is a self-limiting disease with no evident response to anti-bacterial treatment [6]; thus, evaluating the underlying pathology of hip pain in order to exclude serious diagnoses, is of significant importance in the outcome of the disease, since the symptoms of transient synovitis resolve without any treatments within days, while a late treatment of septic arthritis leads to irreversible damage. Septic arthritis is a challenging diagnosis that commonly relies on laboratory tests. Evaluation of joint fluid white blood cell count (WBC) is the best diagnostic test for septic arthritis [7]. However, further diagnostic measures are needed to increase the validity of the currently available methods of differentiating transient synovitis from septic arthritis. C-reactive protein (CRP), erythrocyte sedimentation rate (ESR), and the hip joint effusion in ultrasound have been recommended for arriving at a more definite diagnosis [8]. Few ultrasound features of the hip joint have been identified as distinguishing factors between septic arthritis and transient synovitis. For instance, increased synovial thickness relative to effusion thickness at the ultrasonographic view of the anterior femoral recess has been found to be indicative of septic arthritis [9]. However, no study has evaluated the predictive value of ultrasonographic estimations of hip articular fluid volume in differentiating septic arthritis.

Since these laboratory tests aren’t always readily available, usage of joint aspiration under ultrasound guidance can be used to differentiate septic joints and transient synovitis [10]. High WBC count as well as low fluid glucose increases the likelihood of septic joints [11] and synovial fluid cultures are positive in most septic arthritis cases [12]. Even though it does not always yield a definite result, needle aspiration of the hip joint is the most specific test in diagnosing septic arthritis [13]; however, it is an invasive procedure with itself limitations.

Therefore, to reduce the need for joint aspiration and non-invasive management of patients, we compared the combination of ultrasound findings, and especially ultrasound estimation of hip joint fluid volume, with laboratory findings to assess differentiation septic arthritis from reactive arthritis.

## Methods

### Study settings and approval

Children under the age of 14 who were referred to the Akbar pediatric hospital in 2020–2022 with hip joint pain or limping complaints were included in this longitudinal study according to the inclusion and exclusion criteria. Before participating in this research, all of the required procedures and actions were described to the parents and written consent was obtained. The Ethics Committee of Mashhad University of Medical Sciences approved this study with the approval code of IR.MUMS.MEDICAL.REC.1398.892.

### Participants

The participants were recruited using a non-probability convenience sampling method. In the time extent of this study, 115 patients were referred to the radiology department and entered for the study. The inclusion criteria were as follows; the age of less than 14 years, acute unilateral hip joint effusion, available results of joint effusion aspiration, or arthrotomy, or follow-up. Since purulent arthritis in both hip joints simultaneously is rare, the bilateral hip joint effusion, and the cases where the children or their parents refused more management or follow-up were excluded from the study.

### Data collection

All of the participants underwent bilateral hip ultrasound examinations with a 5–12 MHz linear transducer by a pediatric radiologist using the GE Voluson E6 (Unites states) or Esoate class C (Italy).

**Fig. 1:**
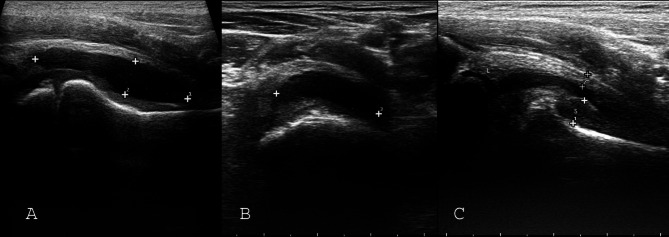
The sonographic measurements of hip effusion in anterior recess: (**A**) Longitudinal diameter and thickness of hip effusion. (**B**) Transverse diameter of anterior recess. The anterior recess volume was measured with these three dimensions. (**C**) Capsule thickness at the midpoint of the anterior recess and synovial thickness at the maximal point. Synovial thickening in reactive arthritis is obvious. L: Labrum, S: Synovium

Hip ultrasound was performed in the supine position and the legs in a neutral position (extension and slight external rotation). Joint effusion was diagnosed from an anterior approach along the long axis of the femoral neck in the parasagittal plane. After confirming an effusion, various ultrasound features of anterior joint recess in longitudinal and transverse views of femoral neck were documented: debris, septated effusion, capsule rupture, dimensions of effusion, effusion volume, and capsule thickness, synovial thickness, total joint thickness, and the difference between the total joint thickness of the healthy and involved joints (Fig. 1).

In all patients, a blood sample was analyzed for erythrocyte sedimentation rate (ESR), C-reactive protein (CRP) level, and WBC counts and glucose. Dependent on patient status and clinical judgment, one of the following three approaches is performed; close follow-up and observation, ultrasound-guided aspiration of the hip joint effusion, and or arthrotomy. The closed follow-up was proposed in the volume of less than 0.5 cc with normal clinical and laboratory examination. An effusion aspiration or arthrotomy of hip joint was performed in other patients. The hip joint puncture and effusion aspiration was carried out under ultrasound guidance in a parasagittal plane after topical anesthesia (lidocaine-prilocaine topical cream) and in a sterile environment. The effusion volume was recorded and the sample was sent to laboratory for: cell count (RBC, WBC, neutrophil), glucose level, lactate dehydrogenase test (LDH), and joint fluid culture. Arthrotomy was performed in patients with unsuccessful aspiration, septated effusion, and septic arthritis.

The presence of a neutrophil ratio of more than 75% with a low joint fluid white blood cell (WBC) count can be suggestive of septic arthritis, even in cases where the culture is negative. Septic arthritis is an infection of the joint, and a high neutrophil ratio along with an elevated WBC count in the joint fluid are indicators of an inflammatory response to infection. However, it is important to note that a negative culture does not completely rule out septic arthritis, as there can be various reasons for false-negative results. Therefore, clinical judgment and further diagnostic tests may be necessary to confirm or exclude the diagnosis. In addition, positive Gram stain in joint aspiration culture, a joint fluid WBC count of more than 50,000 per ml, a neutrophil ratio of more than 75%, joint fluid glucose levels less than 30% of serum glucose levels, and obvious pus were indicative of septic arthritis. The absence of any of these conditions led to the diagnosis of reactive arthritis.

### Statistical analysis

All data was input into the SPSS version 26 software for Windows (IBM Statistics, Chicago, IL) and SAS for analysis. The Kolmogorov-Smirnov test was used to analyze the normal distribution of the data. Quantitative variables were described using mean and standard deviation, while qualitative data were described via tables’ Chi-square test and independent samples t-test were used to compare variables between groups. Correlations between variables were analyzed using Pearson’s correlation test. A p-value less than 0.001 was considered significant. The agreement between ultrasound and aspiration findings was evaluated using interclass correlation coefficient (ICC) and Bland-Altman plotting. A logistic regression model was designed for investigating the odds ratio of differential diagnosis factors between septic arthritis and reactive arthritis. Additionally, a 95% confidence interval was reported for the correlation analyses. Receiver operating characteristic (ROC) curves were employed in order to evaluate the probability of septic arthritis diagnosis for every factor. Along with the ROC curves, the Youden index was used to find the optimal cut-off point for achieving the ideal diagnostic sensitivity and specificity in every analyzed variable. The alpha and beta errors were defined as 0.05 and 0.2, respectively.

## Results

In total, 115 participants were included in this study, 46 of which were females (40.0%). The age of the participants ranged from 1 to 14 years with a mean age of 3.43 ± 5.74. According to articular aspiration and blood analysis, 23 (20.0%) patients were diagnosed with septic arthritis, while the remaining 92 (80.0%) patients were diagnosed with reactive arthritis.

Effusion volume of the hip joint is increased with age: in anterior recess, the estimated mean volume of fluid measured was 0.9 ± 1.1 cc in children less than three years of age, 1 and 4 ± 4 cc in children aged 7 to 14 years. The mean volume of aspirated fluid was 2 ± 1.25 cc in children less than three years old, 5 ± 10.5 cc in children 3 to 6 years old, and 7 ± 7.5 cc in children 7 to 14 years old. According to the Pearson linear correlation analysis, anterior recess effusion volume demonstrated a notable correlation with the measured aspiration fluid volume (r = 0.855, P = 0.003). The intra-class correlation coefficient (ICC) for these variables was r = 0.829 in a 95% confidence interval of 0.754 to 0.883, which indicated a significant agreement between the two (P < 0.001). The mean volume of aspirated fluid was 2.5 times the volume of fluid measured in the anterior recess. In the Bland-Altman plot used to describe the correlation of ultrasound effusion volume with the aspiration fluid volume, most points were between the upper and lower limits and within the mean range, indicating a great correlation between the variables (Fig. 1).

The effusion volume in patients with reactive arthritis and septic arthritis was 2.7 ± 0.5 cc and 2.9 ± 1.7 cc, respectively, without significant difference (P = 0.233).

**Fig. 2:**
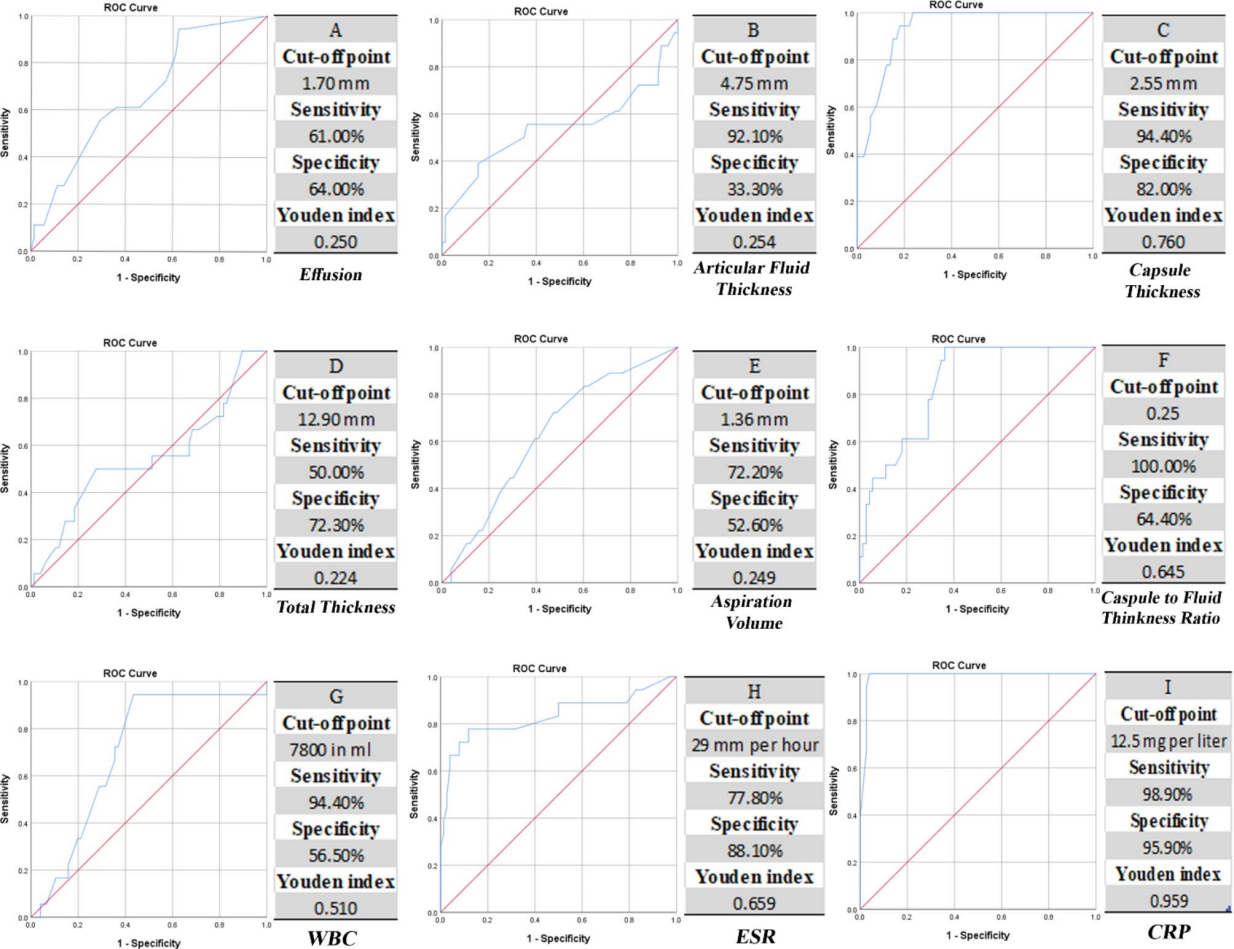
ROC curves and optimum cut-off points for various parameters based on Youden index. (**A**) Effusion volume based on ultrasound. (**B**) Articular fluid thickness based on ultrasound. (**C**) Capsule thickness based on ultrasound. (**D**) Total thickness based on ultrasound. (**E**) Aspiration volume based on ultrasound. (**F**) Capsule thickness to articular fluid thickness ratio based on ultrasound. (**G**) Blood WBC count. (**H**) Blood erythrocyte sedimentation rate (ESR). Blood C-reactive protein level (CRP)

A comparison between the ultrasound findings of the two groups is provided in Table 1. The synovial thickness in reactive arthritis was higher than in septic arthritis (1.83 ± 0.7 mm vs. 2.55 ± 0.9 mm), without significant difference (p = 0.262). There was not a significant difference between the two groups in age, gender, effusion thickness, articular fluid volume, effusion volume, capsule thickness, total thickness, and capsule-to-fluid thickness ratio (P > 0.001). Overall, these results didn’t support the use of hip ultrasound alone as a diagnostic tool for differentiating septic arthritis from reactive arthritis. Figure 2 shows employed ROC curves for evaluation the probability of septic arthritis diagnosis for every factor.

**Table 1:** Comparison of baseline features and clinical observation between septic arthritis and reactive arthritis groups

Feature		Septic arthritis N = 23	reactive arthritis n = 92	p value
**Age (years)**		5.12 ± 4.6	4.63 ± 3.1	0.565
**Gender**	**Female**	13 (56.5%)	45 (48.3%)	0.294
	**Male**	10 (43.5%)	48 (51.6%)	
**CRP (mg/l)**		41.38 ± 39.55	5.96 ± 4.45	0.001
**ESR (mm/h)**		55 ± 14	17.5 ± 2.7	0.000
**WBC (cells per mm)**		7846 ± 2092	8283 ± 3018	0.200
**Synovial thickness (mm)**		1.83 ± 0.7	2.55 ± 0.9	0.262
**Capsule thickness (mm)**		2.8 ± 0.7	2.9 ± 0.8	0.183
**Effusion thickness (mm)**		7.15 ± 2.15	7.0 ± 3.2	0.036
**Total thickness (mm)**		10 ± 2.6	9.6 ± 2.7	0.480
**Differences with opposite side**		4.5 ± 2	3.4 ± 2	0.139
**Effusion volume (mm)** **(Anterior recess)**		2.9 ± 1.7	2.66 ± 0.47	0.233
**Effusion volume (mm)** **(Aspirated volume)**		2.96 ± 0.5	5.6 ± 0.28	0.392

A significant correlation was found between serum ESR, and CRP levels with septic arthritis (P < 0.001). The diagnostic value of the variables in this study was analyzed using logistic regression ROC curves and optimal cut-off points were calculated based on the Youden index while the corresponding sensitivity and specificity values were documented. Between all variables, erythrocyte sedimentation rate (ESR) and CRP levels, demonstrated the greatest sensitivity (sensitivity 82%, specificity 80%) at a cut-off point of 29 while CRP levels showed the greatest sensitivity (97% sensitivity and 94% specificity at a cut-off point of 12.5 mg/l).

**Fig. 3:**
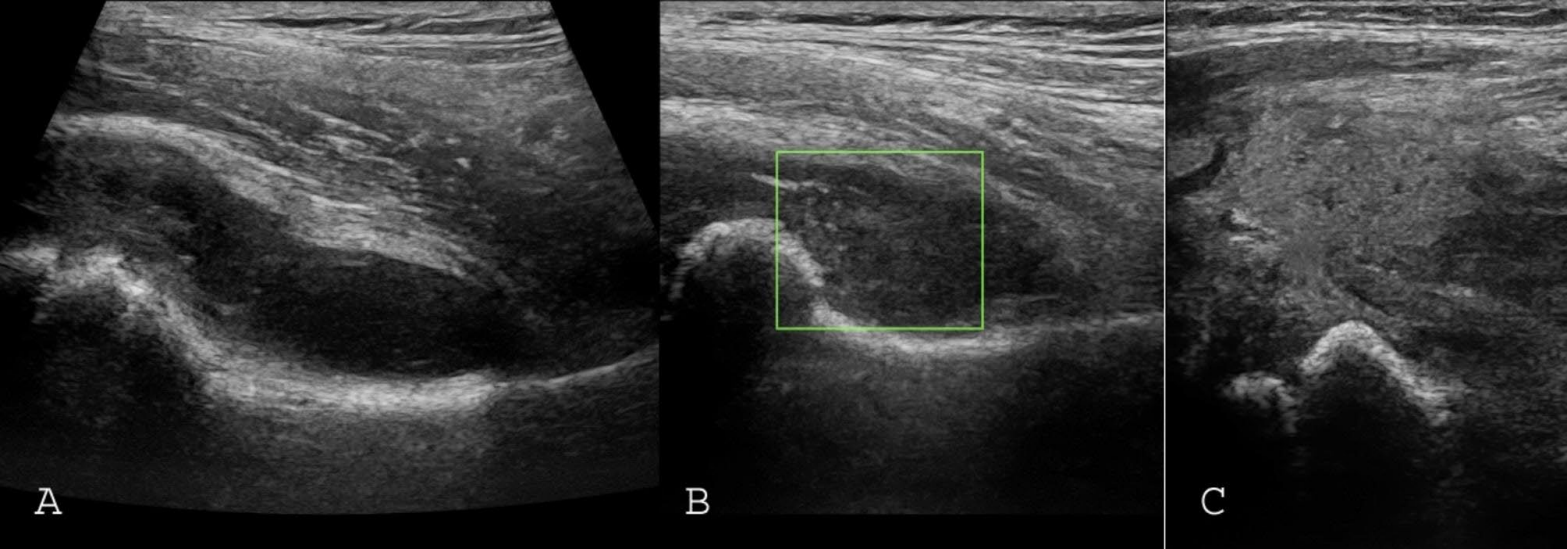
The sonographic sign in three patients with late diagnosed septic arthritis: (**A**) Septated effusion. (**B**) Hip effusion with massive debris (no-vessels on Doppler color box differentiates it from synovial hypertrophy) (**C**) Joint capsule perforation and pus extension to extra- arthicular space

Septic arthritis was not observed in any of the patients with effusion volume less than 0.5 cc and ESR less than 40 or CRP less than 15. 29.5% of our patients had these conditions. The septated effusion and capsule rupture were found in three cases with septic arthritis (Fig. 3).

## Discussion

A proper distinction between self-limiting transient synovitis and more severe septic arthritis is a necessity when dealing with painful hip joint complaints. A decision is often made by analyzing laboratory synovial fluid aspirate findings and blood examinations as well as plain radiographs for ruling out other causes such as fractures or slipped capital femoral epiphysis [14, 15]. Even after obtaining blood tests (WBC, RBC, CRP, and ESR), synovial fluid aspiration remains the gold standard for diagnosing septic arthritis [16, 17]. However, finding other clinical, imaging and laboratory parameters with sufficient sensitivity and specificity could reduce the requirements for synovial fluid aspiration or magnetic resonance imaging (MRI), which are invasive and use anesthesia or sedation. Given the importance of distinguishing septic arthritis, multiple studies have tried to introduce multivariate clinical indicators for such a diagnosis.

The most commonly reported symptom is joint pain, which is found in 85% of septic arthritis patients, followed by joint swelling (in 78%), joint tenderness (up to 100% sensitive), fever of > 39 °C (in up to 58% patients) [12, 18, 19]. Some reports have suggested that high fever is a reliable factor for septic arthritis, since 90% of all patients have a fever of > 37.5 °C [20].

In 1999, Kosher et al. identified 4 predicting factors as a part of a clinical diagnostic algorithm: (A) history of fever, (B) non-weight bearing, (C) ESR > 40ml/h, and (D) WBC count > 12,000 per ml. Kosher stated that the presence of all 4 factors indicates a 93.1% probability of septic arthritis [21]. The idea was later challenged when other studies found contradicting results, a 2004 study for instance, found Kocher’s criteria to be only 59% predictive and suggested 3 other variables: serum WBC count > 12,000 per ml, history of fever and history of related clinical visit [22]. A prospective study in 2006, introduced five similar risk factors identified in their univariate analysis: CRP level > 2.0 mg/l, serum WBC count > 12,000 per mm3, non-weight bearing, ESR > 40 mm/h, and fever. Patients with five predictors demonstrated a 98% chance of septic arthritis and those with four demonstrated a 93% chance. They reported serum CRP level as the strongest independent indicator of septic arthritis [23]. In a similar study, 33 patients who were referred for hip pain were diagnosed with septic arthritis or transient synovitis. Diagnostic factors in favor of septic arthritis were found to be body temperature of more than 38.5 °C, serum CRP level of greater than 10 mg/l [9]. Similarly, our results indicate the diagnostic value of serum ESR and CRP level as an independent predictor of septic arthritis.

It is notable that the ultrasound exam was found to be an optimal diagnostic tool for confirming or excluding effusion [24]. The use of ultrasound as a diagnostic measure for septic arthritis is not a new suggestion. In 1997, the viability of such measurements was investigated in suspected septic arthritis patients with a mean age of 7 years. Mnif et al. found the mean width of the anterior synovial recess to be 11.7 mm (5–20 mm) in the affected hip as opposed to 3.6 mm (2.6-5 mm) in the other healthy hip. They concluded that ultrasound is a highly accurate tool in the early diagnosis of joint effusion, while the hyperechoic or mixed aspect of the joint fluid is suggestive of septic arthritis [25]. However, some reports had suggested that ultrasound is not a safe modality for distinguishing septic arthritis from transient synovitis. For instance, a 2006 study concluded that even though ultrasound is a viable noninvasive modality for the detection of hip effusion, it does not aid in the differential diagnosis between septic arthritis and transient synovitis, as to avoid the need for aspiration or anesthesia. The clinical outcome was reported unsatisfactory for four out of eight patients who had false-negative ultrasound results [24].

Chin et al. (2017). assessed the capsule thickness relative to the effusion thickness at the femoral recess; Predominant synovial (capsular) thickening relative to joint effusion thickness at the anterior femoral recess had a significant relation with reactive arthritis (p = 0.012). ROC curves for joint effusion thickness demonstrated a respective sensitivity and specificity of 71% and 56% at a cut-off point of 7.5 mm, therefore suggesting it is a satisfactory diagnostic factor for septic arthritis. They ultimately concluded that ultrasound can aid in predicting septic arthritis when predominant capsule thickening relative to joint effusion and increased thickness of the anterior femoral recess and joint effusion thickness > 7.5 mm should warn the physician of a possibility of septic arthritis [9]. In our study, although the synovial thickness in reactive arthritis was higher than in septic arthritis (1.83 ± 0.7 mm vs. 2.55 ± 0.9 mm), but we didn’t find a significant difference between them (p = 0.262). In addition, there was not a significant difference between septic arthritis and reactive arthritis in age, gender, effusion thickness, capsule thickness, total articular space thickness. A significant correlation was only found between serum ESR and CRP levels, and septic arthritis. Multi-variate logistic regression showed ESR and CRP as independent predictors of septic arthritis (P ≤ 0.001).

Our results show the mean volume of aspirated fluid was 2 ± 1.25 cc in children less than three years old, 5 ± 10.5 cc in children 3 to 6 years old, and 7 ± 7.5 cc in children 7 to 14 years old. There was a notable correlation between volume of fluid measured in anterior recess and the aspirated volume. The mean volume of aspirated fluid was 2.5 times the volume of fluid measured in the anterior recess. These results can help to choose the size of the syringe for aspiration. Although, our results absolutely didn’t support the use of effusion volume as an indication of septic arthritis, but they show that septic arthritis was not observed in any of the patients with effusion volume less than 0.5 cc and ESR less than 40 or CRP less than 15. Then conservative management and close follow-up can be recommended in about 30% of patients (effusion volume < 0.5 cc and natural inflammatory factors).

As some have suggested that ultrasound may demonstrate a low sensitivity and specify in early assessments of hip joint condition (24), it has also been suggested that physicians should be cautious when evaluating ultrasound scans, taken within 24 h of initial symptoms [26].

The limiting factor of this study was that the interval between the onset of symptoms and the ultrasound examination was not considered as a variable. While our research has this limiting factor, future investigations are recommended to support the use of ultrasound as an early diagnostic modality for septic arthritis. Additionally, these results with the clinical findings as an alternative to aspiration need to be evaluated in order to support its use.

## Conclusion

The average volume of aspirated fluid was 2.5 times the volume of fluid measured in the anterior recess of hip. Since septic arthritis was not observed in any of the patients with effusion volume < 0.5 cc and normal inflammatory factors, conservative management and close follow-up can be recommended in these patients instead of joint fluid aspiration.

### Electronic supplementary material

Below is the link to the electronic supplementary material.

Supplementary Material 1

## Data Availability

The data and materials used in this study are available upon request from the corresponding author.

## Declarations

### Constant of publication

This manuscript has not been published elsewhere and is not under consideration for publication in any other journal.

## Abbreviations

WBC: White blood cell count
ESR: Erythrocyte sedimentation rate
CRP: C-reactive protein
MRI: Magnetic resonance imaging
ICC: The intra-class correlation coefficient
ROC: Receiver operating characteristic
SPSS: Statistical Package for the Social Sciences
SAS: Special Air Service
