# Supplementary material for: Role of ultrasound and inflammatory factors in the management of pediatric hip joint effusion
Source: Pediatr Rheumatol Online J. 2023 Dec 19;21:146. doi: 10.1186/s12969-023-00922-8 (PMC10729365; doi:10.1186/s12969-023-00922-8)
Supplement: Supplementary file 1 — Supplementary Material 1 [file 12969_2023_922_MOESM1_ESM.docx]

*A clear explanation of these findings should be written.****o What is the difference between debris and organized effusion?***

*Debris: Slightly echogenic tiny particles floating within joint fluid which could be due to pus formation or hemorrhage*

*Septate effusion: Presence of septa within joint fluid also referred to as organizing effusion and is associated with SA*

***o How did you diagnose periosteal reaction by US?***

*We did not measure or diagnose periosteal reaction via ultrasound evaluation.*

***o Why did you examine the capsule rupture?***

*Non traumatic Capsular rupture is an infrequent complication of septic arthritis however it has high specificity and requests emergent arthrotomy. In patients with capsular rupture and pus extension to peripheral extra articular space, arthrocentesis is not indicated.*

***o What is the difference between the dimensions of effusion and effusion thickness?***

*Anteroposterior diameter of hip joint effusion:* Measures as maximum distance between anterior and posterior layers of anterior joint capsule in anterior femoral recess perpendicular to femoral axis on sagittal plane (also referred to as effusion thickness)

***o How did you measure effusion volume since the volume is presented with cm3 and you have presented this in the Figures as mm?***

*effusion volume has been measured in milli liter****o What is the difference between capsule thickness, synovial thickness, and total thickness?***

*The term Synovial thickness has been replaced with anterior layer of anterior capsule thickness.*

*Anterior hip joint capsule is divided into anterior and posterior layers by joint effusion.*

***o What do you mean by anterior recess thickness?***

*Total thickness of hip anterior recess:* Maximum thickness of anterior and posterior layers of anterior hip capsule measure on sagittal plane parallel to femoral axis + anteroposterior diameter of hip joint effusion

**Explain in detail the US technic, and how was the child situated during the US examination. Which probe was used for the small children and which for the older children?**

All of the participants underwent bilateral ultrasound examinations of the hip region by a pediatric radiologist with 10 years of experience using the GE Voluson E6 (GE Medical Systems, Milwaukee, WI, USA) or Esoate class C (Italy) with a 7.5-12 MHz linear transducer. There was no preference in using any of the ultrasound devices for specific group of patients. Patients were examined in supine position with the hips in neutral position (extension and slight external rotation). An anterior approach along the long axis of the femoral neck was used to visualize the anterior capsule of the hip joint to the best advantage .

***Separate the patients into age groups.***

| Age group(years) | No. patients | Effusion volume in anterior femoral recess(cc) | aspirated fluid volume(cc) |
| --- | --- | --- | --- |
| 3> | 30 | 1.1±0.9 | 2±1.25 |
| 3-6 | 47 | 1.4±1.49 | 10.5±5 |
| 7< | 38 | 4±1.4 | 7.5±7 |

**• Define the inclusion criteria more clearly. • Define the inclusion criteria more clearly. You have written that only children with unilateral hip joint effusion and availability results of joint effusion aspiration, arthrotomy or follow-up were included in the study. Why did you include only children with unilateral hip joint effusion? Also, it is not clear to me did you performed joint effusion aspiration on all children or how did you decide to whom joint effusion aspiration will be performed. A clear explanation is needed. What markers were examined in the joint effusion?**

our inclusion criteria were as follows; acute onset mono-articular hip pain, age under 18 years, unilateral hip joint effusion in ultrasound evaluation and availability of laboratory reports for hip joint fluid analysis.

We included patients with unilateral hip joint effusion because we are working on patients with monoarticular presentation. Septic arthritis can be ruled out by confident in patients with more than joint involvement.

Closed follow-up was proposed in the joint effusion volume less than 0.5cc with a normal physical examination and with CBC-diff and inflammatory factors (ESR, CRP) within normal range. Aspiration of hip effusion was proposed in patients who lacked any of the close follow up criteria.

**• Define the exclusion criteria and the number of excluded patients as well**

. We excluded patients with bilateral joint effusion as well as patients presenting with acute oligo-articular and poly-articular symptoms because of not being a good candidate for hip arthrocentesis and having low rate of association with septic arthritis or Reactive arthritis.

In addition, patients with radiography confirmation of having hip or extra-hip problems that mimic hip SA or RA clinically such as acute hip fracture, slipped capital femoral epiphysis or tumor, patients with systemic diseases causing immunosuppression such as malignancies or chronic renal failure, patients with chronic or recurrent arthritis, rheumatologic disease, bone or joint disease and patients taking immunosuppressive medications were also excluded.

**The presence of neutrophil ratio of more then 75% with low joint fluid WBC count was considered suggestive for septic arthritis even in case of negative colture?**

the presence of a neutrophil ratio of more than 75% with a low joint fluid white blood cell (WBC) count can be suggestive of septic arthritis, even in cases where the culture is negative. Septic arthritis is an infection of the joint, and a high neutrophil ratio along with an elevated WBC count in the joint fluid are indicators of an inflammatory response to infection. However, it is important to note that a negative culture does not completely rule out septic arthritis, as there can be various reasons for false-negative results. Therefore, clinical judgment and further diagnostic tests may be necessary to confirm or exclude the diagnosis
